# Encapsulating Metal-Organic-Framework Derived Nanocages into a Microcapsule for Shuttle Effect-Suppressive Lithium-Sulfur Batteries

**DOI:** 10.3390/nano12020236

**Published:** 2022-01-12

**Authors:** Jinyun Liu, Yajun Zhu, Junfei Cai, Yan Zhong, Tianli Han, Zhonghua Chen, Jinjin Li

**Affiliations:** 1 Anhui Provincial Engineering Laboratory for New-Energy Vehicle Battery Energy-Storage Materials, College of Chemistry and Materials Science, Anhui Normal University, Wuhu 241002, China; yjzhu@ahnu.edu.cn (Y.Z.); yanzhong@ahnu.edu.cn (Y.Z.); hantianli@ahnu.edu.cn (T.H.); 2 National Key Laboratory of Science and Technology on Micro/Nano Fabrication, Department of Micro/Nano-Electronics, Shanghai Jiao Tong University, Shanghai 200240, China; caijf001@sjtu.edu.cn; 3 Shenzhen FBTech Electronics Ltd., Shenzhen 518100, China

**Keywords:** secondary battery, nanocomposite, microcapsule, capacity, stability

## Abstract

Long-term stable secondary batteries are highly required. Here, we report a unique microcapsule encapsulated with metal organic frameworks (MOFs)-derived Co_3_O_4_ nanocages for a Li-S battery, which displays good lithium-storage properties. ZIF-67 dodecahedra are prepared at room temperature then converted to porous Co_3_O_4_ nanocages, which are infilled into microcapsules through a microfluidic technique. After loading sulfur, the Co_3_O_4_/S-infilled microcapsules are obtained, which display a specific capacity of 935 mAh g^−1^ after 200 cycles at 0.5C in Li-S batteries. A Coulombic efficiency of about 100% is achieved. The constructed Li-S battery possesses a high rate-performance during three rounds of cycling. Moreover, stable performance is verified under both high and low temperatures of 50 °C and −10 °C. Density functional theory calculations show that the Co_3_O_4_ dodecahedra display large binding energies with polysulfides, which are able to suppress shuttle effect of polysulfides and enable a stable electrochemical performance.

## 1. Introduction

Recently, the demands for electric vehicles and portable electronics have been rapidly increasing. Considering this, investigations on secondary batteries are considered to be a significant direction. People are expecting higher capacity, longer cycling life, and faster charging of secondary batteries. As a promising next-generation secondary battery, the Li-S battery possesses a high theoretical energy density (2600 Wh kg^−1^) and low cost of sulfur [1,2,3,4]. Recently, the research on Li-S batteries has been considered to be a significant field [5,6]. There are many problems for currently available Li-S batteries, such as the volumetric change of sulfur and shuttle effect reducing the electrochemical performance, which represent obstacles to commercialization [7].

In order to address those issues, many studies have been reported, in which the synthesis of yolk-shell structure hosts is considered to be a potential strategy [8,9,10]. Zhang et al. reported a yolk-shell ZnO by using a hydrothermal method, which provided a specific capacity of 1406 mAh g^−1^ at 0.1C [11]. Jiang et al. synthesized a yolk-shell nanomaterial consisting of SiO_2_ core and carbon shell [12]. The cathode based on the yolk-shell SiO_2_-carbon delivered 1200 mAh g^−1^ at a rate of 0.2C. Those achievements indicate that several yolk-shell structures exhibit enhanced adsorption towards polysulfides [13,14]. Reasonable engineering yolk-shell structure as sulfur host could promisingly improve the energy-storage properties [15,16]. However, general and simple preparation approaches for large-scale yolk-shell materials are still highly required. In addition, several reports indicated that typical semiconductor Co_3_O_4_ is a promising sulfur host because of its strong interaction with sulfur and polysulfides [17,18,19,20]. With this in mind, developing creative Co_3_O_4_-based composite for Li-S batteries is attractive and would be of great significance [21,22,23].

**Figure 1:**
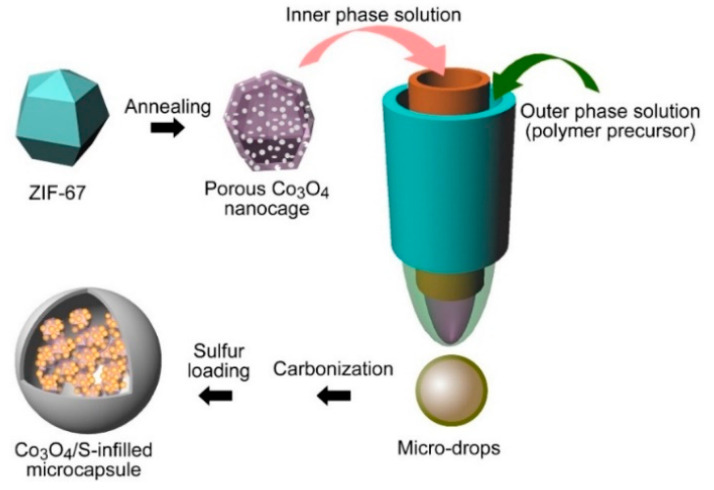
Microfluidic preparation of Co_3_O_4_/S-infilled microcapsules.

Here, we present a microcapsule infilling by a metal–organic framework (MOF)-derived cobalt oxide nanocage as the sulfur host, which displays a high electrochemical performance. By using ZIF-67 as a precursor which was prepared through a hydrothermal approach, a porous dodecahedral Co_3_O_4_ nanocage was obtained (Figure 1). The experimental procedures are presented in the Supplementary Material. Then, Co_3_O_4_ nanocages were encapsulated into microcapsules through a microfluidic strategy. The real-time process of the cone and the formation of drops are displayed by Movie S1 and S2, respectively. The prepared microcapsules were carbonized for use as a sulfur host, which showed a long life of 200 cycles, along with a stable specific capacity of 935 mAh g^−1^ and a 100% Coulombic efficiency. After repeated tests, it displays a stable rate-performance, and the battery remains stable at −10 °C and 50 °C. Furthermore, density functional theory (DFT) calculations show that Co_3_O_4_ possesses large binding energies towards polysulfides, which are important for reducing the shuttle effect and enabling a stable electrochemical performance.

## 2. Results and Discussion

### 2.1. Structural and Microstructural Characterization

**Figure 2:**
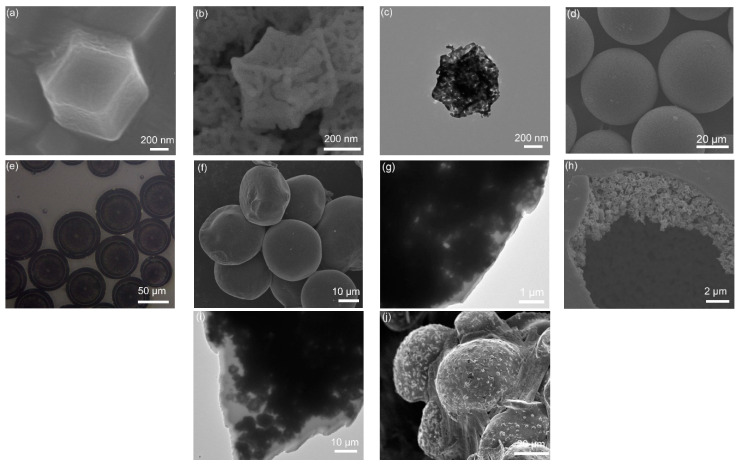
SEM photographs of (**a**) ZIF-67 and (**b**) dodecahedral Co_3_O_4_ nanocage. (**c**) TEM image of Co_3_O_4_. (**d**) SEM and (**e**) optical photographs of Co_3_O_4_-infilled microcapsules before carbonization. (**f**) SEM and (**g**) TEM images of carbonized microcapsules. (**h**) SEM and (**i**) TEM photographs of Co_3_O_4_-infilled microcapsule after breaking manually. (**j**) SEM image of microcapsules after loading sulfur.

SEM image (Figure 2a) of ZIF-67 precursor shows a dodecahedron morphology with a size of 500 nm. Figure 2b shows the SEM image of porous dodecahedral Co_3_O_4_ obtained after annealing the precursor. The TEM image (Figure 2c) displays the porous Co_3_O_4_ nanocage clearly. The dodecahedral Co_3_O_4_ was encapsulated in microcapsule by using a coaxial focusing method. Figure 2d shows the SEM image of microcapsules with a size of about 50 μm. The microcapsule was observed by using an optical microscope (Figure 2e), and it is verified the Co_3_O_4_ uniformly distributes in the microcapsule. An SEM image of Co_3_O_4_-infilled microcapsules after annealing is shown in Figure 2f. A TEM image (Figure 2g) presents the edge of the microcapsule. It is observed that the shell of the microcapsule is very thin after calcination. After the microcapsules were broken manually, dodecahedral Co_3_O_4_ nanocages inside the microcapsule were observed clearly in Figure 2h,i. Figure 2j displays the microcapsules after loading sulfur, forming a Co_3_O_4_/S-infilled microcapsule structure. The surface become rough, which indicates that some of the sulfur was coated on microcapsules.

**Figure 3:**
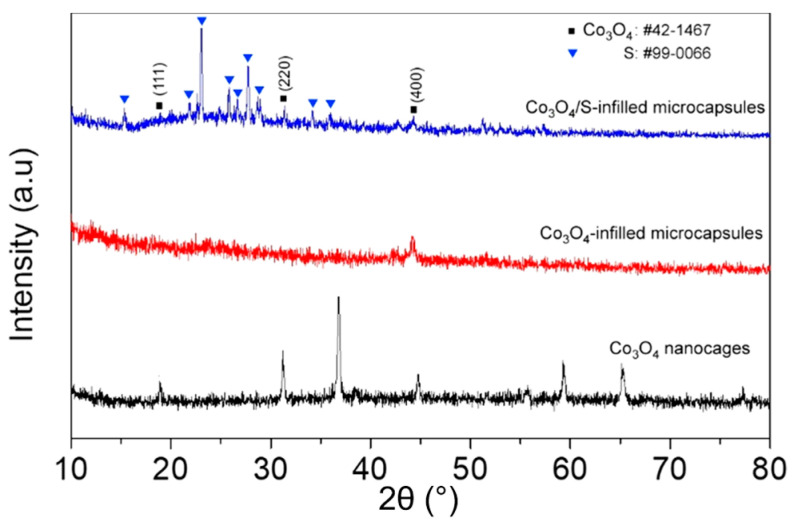
XRD patterns of the pristine Co_3_O_4_ nanocages and the capsules infilled with Co_3_O_4_ or Co_3_O_4_/S composite.

The XRD pattern (Figure 3) is assigned to Co_3_O_4_ in terms of JCPDS card No. 42-1467, while the other peaks are attributed to sulfur (JCPDS card No. 99-0066). The signal of Co_3_O_4_ becomes unobvious after loading sulfur, which would be ascribed to the cover of sulfur signals. Figure S1 displays the HRTEM image of the porous Co_3_O_4_. The Co_3_O_4_ obtained after annealing ZIF-67 precursor exhibits a good crystallinity. The 0.28 nm lattice spacing matches the (220) crystalline plane, while the SAED pattern displays several diffraction rings, indicating a polycrystalline structure [24].

**Figure 4:**
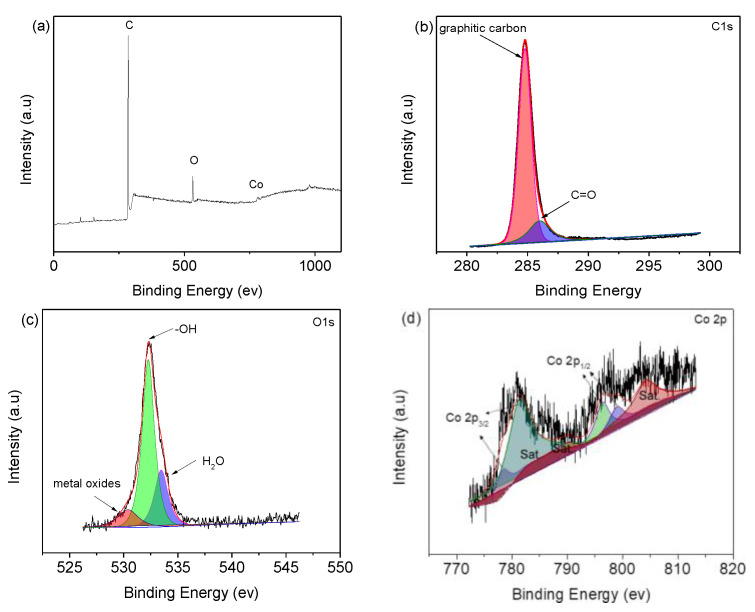
(**a**) XPS survey spectrum of Co_3_O_4_-infilled microcapsules. XPS spectra of (**b**) C 1s, (**c**) O 1s, and (**d**) Co 2p.

The composition and chemical states of the microcapsules are presented in the XPS spectra (Figure 4). The peak at 285 eV is from C1s, and the ones at 530 and 790 eV are ascribed to the O1s and Co2p, respectively, as shown in Figure 4a. The C 1s spectrum (Figure 4b) shows two peaks, where the one at 284.6 eV is indexed to graphite carbon. Moreover, peak at 285.9 eV represents C=O [17]. O 1s spectrum (Figure 4c) exhibits three peaks at 530 eV, 532.2, and 533.5 eV, corresponding to lattice oxygen, ‒OH and H_2_O molecules, respectively [25,26,27]. The Co 2P spectrum (Figure 4d) shows 781.0 and 799 eV of Co^2+^ [28], and 778.7 and 796.6 eV peaks are from Co^3+^ [29,30].

**Figure 5:**
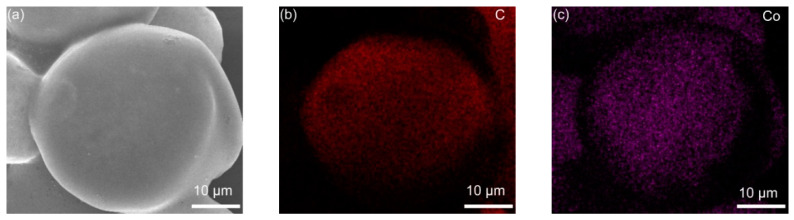

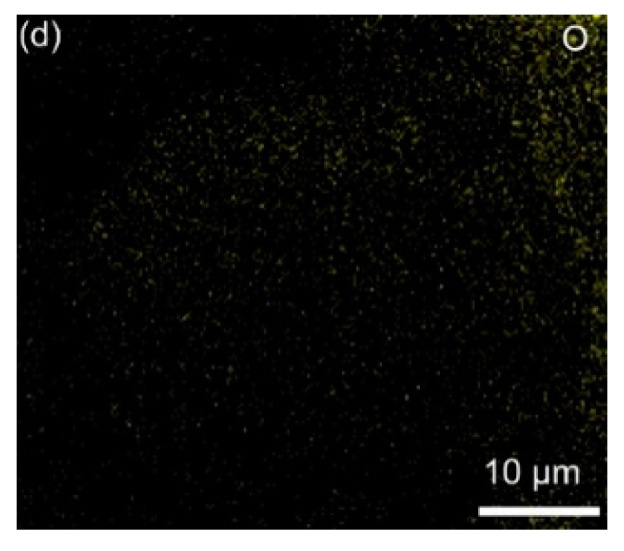
(**a**) SEM and (**b**–**d**) mapping images of the Co_3_O_4_-infilled microcapsules. (**e**) Related EDS spectrum.

**Figure 6:**
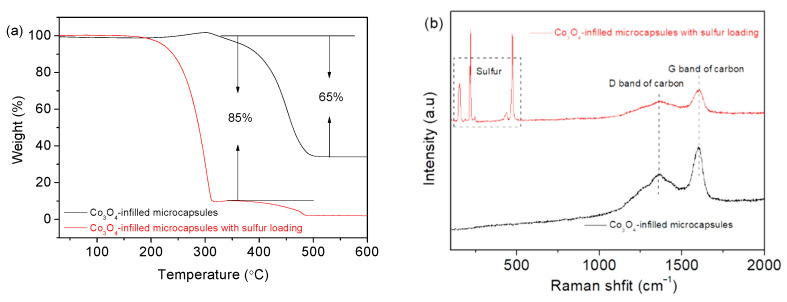
(**a**) TGA profiles and (**b**) Raman spectra of Co_3_O_4_-infilled microcapsules with and without loading sulfur.

The elemental distribution of Co_3_O_4_-infilled microcapsules is shown in Figure 5. The elements C, Co, and O evenly distribute. In Figure 5e, the EDS spectrum shows that the composition of Co_3_O_4_ includes C, Co, and O [31], which has a high purity. Figure 6a presents the TGA curves of Co_3_O_4_-infilled microcapsules measured in air. The mass loss from 350 °C to 480 °C is caused by the decomposition of the carbon shell. The drop from 250 to 350 °C is attributed to sulfur evaporation [32,33]. The sulfur in the microcapsules is about 85 wt%, which is significant for a high-sulfur loading. Moreover, pure Co_3_O_4_-infilled microcapsules show that the content of Co_3_O_4_ nanocages is about 35 wt%. Figure 6b shows the Raman spectra of the Co_3_O_4_-infilled microcapsules with and without loading sulfur. The D- and G-bands of carbon locate at 1370 and 1670 cm^−1^, respectively. In sulfur-loaded microcapsules, the peaks ranging from 150 to 475 cm^−1^ are assigned to sulfur.

### 2.2. Electrochemical Characterization

**Figure 7:**
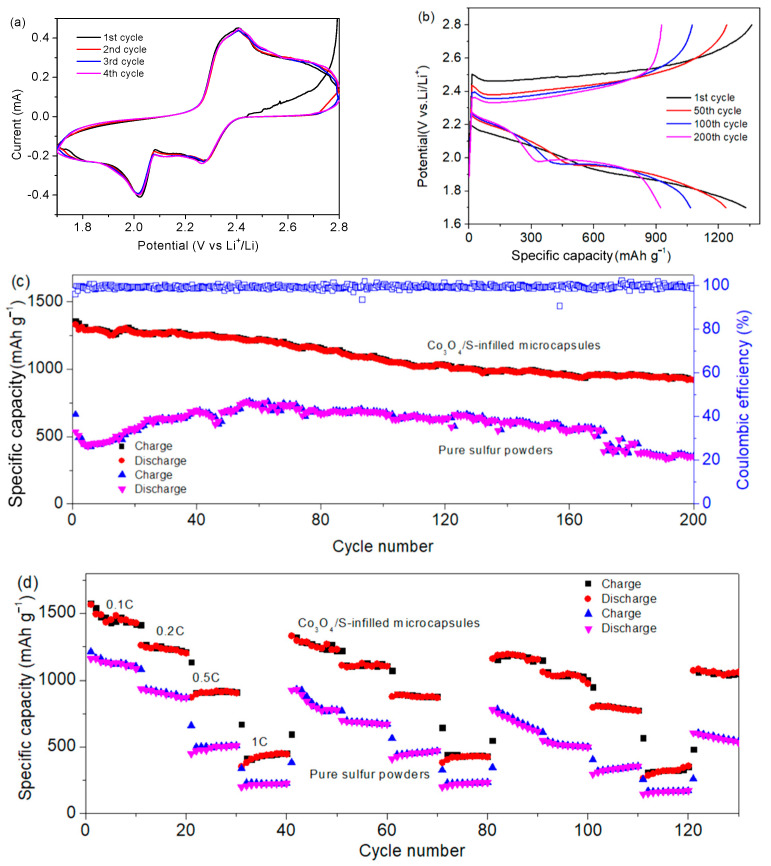
(**a**) CV curves of Co_3_O_4_/S-infilled microcapsules-based Li-S battery scanning at a rate of 0.1 mV s^−1^. (**b**) Cycling curves at 0.5C. (**c**) Cycling performance at a rate of 0.5C. (**d**) Rate-performance of different samples.

Figure 7a displays the CV curves. During the discharge, two reduction peaks at 2.25 and 1.95 V are attributed to the conversion of high-order polysulfide to Li_2_S_4_ and reduction of Li_2_S_4_ to Li_2_S_2_/Li_2_S [34,35]. In the charging process, the oxidation at 2.4 V is ascribed to the conversion of Li_2_S_2_/Li_2_S to Li_2_S_n_ [36]. In Figure 7b, two discharge plateaus at 2.2 and 1.9 V are verified. The platform at 2.4 V in charge corresponds to oxidation peak [37]. It is noted that the overpotentials are observed, which may be attributed to the non-completed carbonization and the resulting limited conductivity. Figure 7c shows that the specific capacity remains 935 mAh g^−1^ after cycling 200 times at 0.5C (1C equates to fully charging or discharging the theoretical capacity in 1 h). The Coulombic efficiency is close to 100%. Compared to the performance of the Co_3_O_4_/S-infilled microcapsules, the capacity of pure sulfur powders is very low. In particular, the capacity decays rapidly after 150 cycles. In addition, the electrochemical performance of the microcapsules is also competitive compared to some other composites, as displayed in Table 1. Figure 7d shows the rate performance after repeated tests. In the second round, specific capacities are 1250, 1150, 860, and 500 mAh g^−1^ at rates of 0.1C, 0.2C, 0.5C, and 1C, respectively. It recovers to 1190 mAh g^−1^ once the rate is returned to 0.1C. Microcapsules exhibit a better reversibility and higher capacities than sulfur powders. It is attributed to the improved conductivity by the carbon shell and the reduced polysulfide loss by Co_3_O_4_ adsorption, which will be demonstrated by DFT calculations.

**Figure 8:**
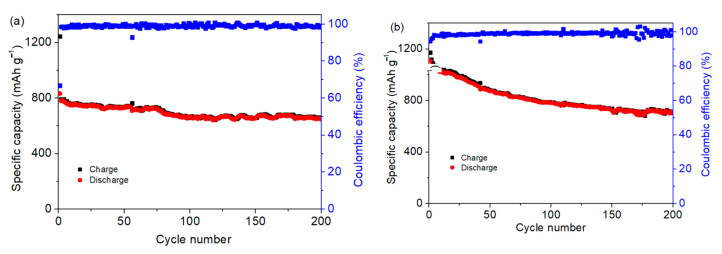
Specific capacities of Co_3_O_4_/S-infilled microcapsules when cycling at different temperatures of (**a**) −10 °C and (**b**) 50 °C at 0.5C.

The Co_3_O_4_/S-infilled microcapsules-based Li-S battery also displays good cycling stability under different temperatures. Figure 8a shows that the capacity remains at 647 mAh g^−1^ after cycling 200 times under −10 °C. Besides cycling at a low temperature, the electrochemical performance at 50 °C is presented in Figure 8b, showing a specific capacity of 713 mAh g^−1^ after 200 cycles. Stable performance indicates that microcapsules can be used in different conditions, which are significant for practical applications.

**Figure 9:**
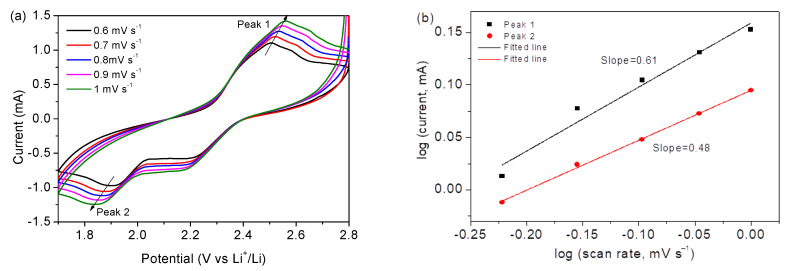

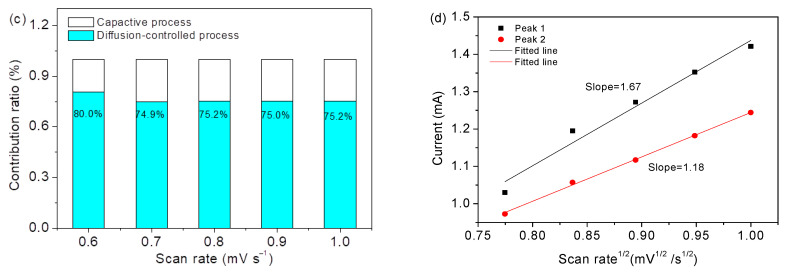
(**a**) CV profiles of the Co_3_O_4_/S-infilled microcapsules. (**b**) Relationship of log(*v*) vs log(*i*). (**c**) Contribution ratios. (**d**) Relationship of peak current vs. rate.

Figure 9a displays CV curves of Co_3_O_4_/S-infilled microcapsules at 0.6 to 1 mV s^−1^; Figure 9b displays a logarithmic relationship according to *i* = a*v*^b^, where *i* and *v* stand for the peak current and rate, respectively [49]. The b value of 0.5 represents a diffusive-controlled process, and b = 1 indicates a capacitive behavior. In this investigation, b values suggest mainly diffusion-controlled processes. Figure 9c shows the diffusion contribution ratios calculated on the basis of *i*(v) = k_1_*v* + k_2_*v*^1/2^, where k_1_*v* and k_2_*v*^1/2^ stand for capacitive and diffusion-controlled contributions, respectively. The results were fitted (Figure 9d) based on *I*_P_ = 2.69 × 10^5^ × n^3/2^AD^1/2^C*v*^1/2^ [50], where *I*_p_ is the peak current; n is the number of electrons transferred during the reaction, which is 2 for Li-S batteries; A is the active electrode area (1.13 cm^2^); D is the diffusion coefficient of lithium ion in unit of cm^2^ s^−1^; C is the concentration of Li ions in electrolyte in unit of mol mL^−1^ [51,52]; and n is the scanning rate in the unit of V s^−1^. On the basis of the obtained slopes, the Li ion diffusion coefficients are calculated to be 3.8 × 10^−9^ and 1.8 × 10^−9^ cm^2^ s^−1^, which are close to some reports [53,54]. The good diffusion property is ascribed to the specific microcapsule structure, which enables a good environment for contact with electrolytes; the porous scaffold of Co_3_O_4_ nanocages assembled by nanoparticles shortens the transfer pathway of ions.

### 2.3. DFT Calculations

**Figure 10:**
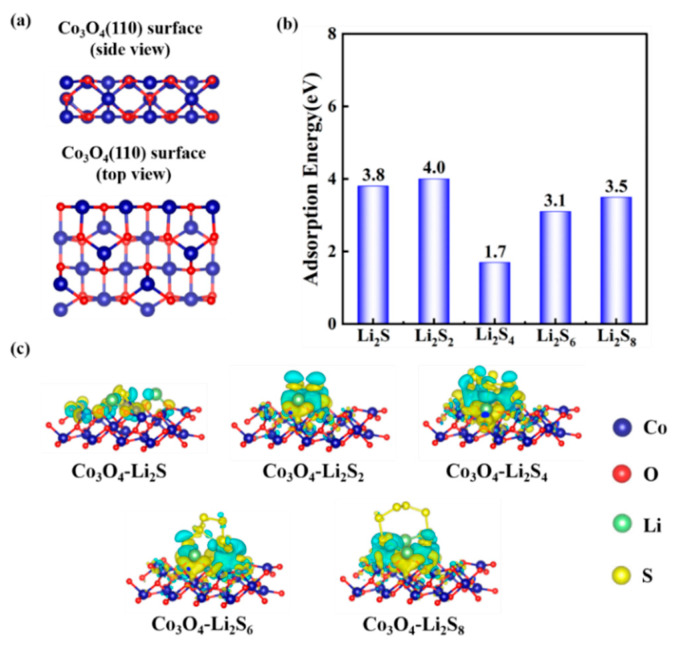
(**a**) Side and top views of the geometric configurations of Co_3_O_4_ (110) surface. (**b**) Surface adsorption energies towards polysulfides. (**c**) Electron density differences of the polysulfides (Li_2_S, Li_2_S_2_, Li_2_S_4_, Li_2_S_6_, Li_2_S_8_).

**Figure 11:**
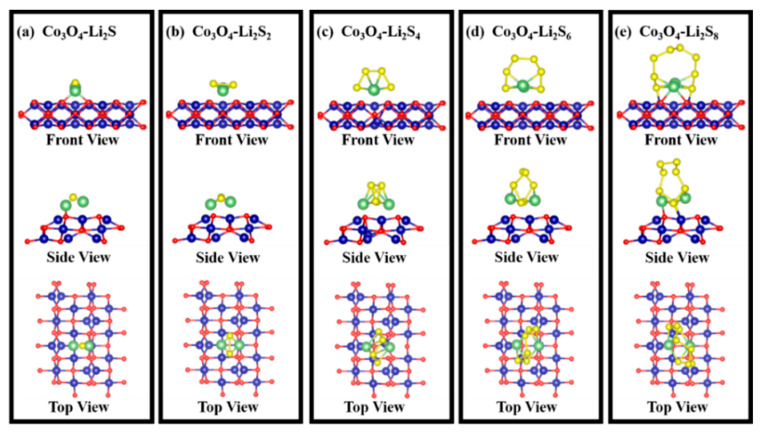
Adsorption models between polysulfides and Co_3_O_4_ (110) surface: (**a**) Co_3_O_4_-Li_2_S; (**b**) Co_3_O_4_-Li_2_S_2_; (**c**) Co_3_O_4_-Li_2_S_4_; (**d**) Co_3_O_4_-Li_2_S_6_; (**e**) Co_3_O_4_-Li_2_S_8_.

The binding of the Co_3_O_4_ with polysulfides was investigated by using DFT calculation on the adsorption energies. All calculations were conducted by using a Vienna Ab initio Simulation Package. In Figure 10, a series of surface adsorptions of Co_3_O_4_ towards the polysulfides (Li_2_S, Li_2_S_2_, Li_2_S_4_, Li_2_S_6_, Li_2_S_8_) are presented. According to previous research [55,56,57], the (110) lattice plane in the Co_3_O_4_ is more prone to exposure due to the presence of Co^3+^ on the surface. Therefore, we focused on the adsorption energy of the polysulfides on the Co_3_O_4_ (110) surface. The side and top views of the geometric configurations of Co_3_O_4_ (110) surface are shown in Figure 10a. Then, the adsorption models were built up for the calculation of adsorption energy, which is shown in Figure 11. Figure 10b shows the surface adsorption energies toward the polysulfides, where the surface adsorption energies of the Co_3_O_4_ toward Li_2_S, Li_2_S_2_, Li_2_S_4_, Li_2_S_6_, Li_2_S_8_ are 3.8, 4.0, 1.7, 3.1, and 3.5 eV, respectively, indicating a good adsorption capability of Co_3_O_4_ towards polysulfides. Figure 10c displays the charge density difference of the adsorption models. The distribution of the charge density connects polysulfides and Co_3_O_4_, indicating an electron transfer between Co_3_O_4_ (110) surface and polysulfides. The charge density between polysulfides and Co_3_O_4_ illustrates the formation of bonds and further verifies the adsorption stability.

## 3. Conclusions

In summary, a novel microcapsule system encapsulated with MOFs-derived Co_3_O_4_/S nanocages is developed, which displays a good electrochemical performance as a Li-S battery cathode. Dodecahedral ZIF-67 was synthesized, then it was converted to a porous Co_3_O_4_ nanocage which was infilled into a microcapsule through a microfluidic strategy. After 200 cycles at 0.5C, the specific capacity of Co_3_O_4_/S-infilled microcapsules remains 935 mAh g^−1^. The Coulombic efficiency is about 100%. The constructed battery also shows a stable rate-performance, while good capacities are also achieved under both high and low temperatures of 50 °C and −10 °C. In addition, DFT calculations verify that the Co_3_O_4_ displays large binding energies towards all polysulfides including Li_2_S, Li_2_S_2_, Li_2_S_4_, Li_2_S_6_, and Li_2_S_8_, reducing the loss of polysulfides. It is expected that the developed microcapsule system and the high performance will be applicable for engineering other emerging Li-storage nanomaterials.

## Figures and Tables

**Table 1:** Comparison on the electrochemical performance of some composite-based cathodes.

Composite	Preparation Method	Cycling Rate	Cycle Number	Specific Capacity (mAh g^‒1^)	Ref.
TiO_2_@Co_3_O_4_/S nanospheres	Water bath	0.1C	100	817	[38]
Co_3_O_4_ powders/S	Hydrothermal method	0.1C	100	706	[39]
Co_3_O_4_ -CoN/CC	Hydrothermal method	3C	500	627	[40]
Co_3_O_4_/CoO/GNS/h-BN/S	Ball-milling	1C	250	356	[41]
S@Co_3_O_4_/C	Hydrothermal method	1C	500	520	[42]
Nano S/rGO	High pressure steam	5C	100	639	[43]
NiCo_2_S_4_@S	Hydrotherma method	0.5C	500	836	[44]
Yttria hollow spheres@C/S	Hydrothermal method	0.5C	200	842	[45]
Mo@N-G/S	Hydrothermal method	1C	500	615	[46]
S@N-Ta_2_O_5_/rGO	Co-precipitation	2C	600	825	[47]
S@MnO_2_@SnO_2_	Hydrothermal method	0.5C	500	566	[48]
Co_3_O_4_/S-infilled microcapsules	Microfluidic approach	0.5C	200	935	This work

## Data Availability

Data sharing not applicable.

## Supplementary Materials

The following supporting information can be downloaded at: https://www.mdpi.com/article/10.3390/nano12020236/s1, Figure S1: (a) HRTEM image and (b) selective area electron diffraction (SAED) pattern of the porous Co_3_O_4_. Movie S1: Real-time process of the cone. Movie S2: Formation of drops.
