# Supplementary material for: Encapsulating Metal-Organic-Framework Derived Nanocages into a Microcapsule for Shuttle Effect-Suppressive Lithium-Sulfur Batteries
Source: Nanomaterials (Basel). 2022 Jan 12;12(2):236. doi: 10.3390/nano12020236 (PMC8777985; doi:10.3390/nano12020236)
Supplement: Supplementary file 1 [file nanomaterials-12-00236-s001.zip › nanomaterials-1505929-SI.pdf]

Supplementary Materials

Encapsulating Metal-Organic-Framework Derived Nanocages into a Microcapsule for Shuttle Effect-Suppressive Lithium-Sulfur Batteries

Jinyun Liu [1,†,*], Yajun Zhu [1,†], Junfei Cai [2,†], Yan Zhong [1], Tianli Han [1], Zhonghua Chen [3,*] and Jinjin Li [2,]

[1] Anhui Provincial Engineering Laboratory for New-Energy Vehicle Battery Energy- Storage Materials, College of Chemistry and Materials Science, Anhui Normal University, Wuhu 241002, China; yjzhu@ahnu.edu.cn (Y.Z.); yanzhong@ahnu.edu.cn (Y.Z.); hantianli@ahnu.edu.cn (T.H.)

[2] National Key Laboratory of Science and Technology on Micro/Nano Fabrication, Department of Micro/Nano-electronics, Shanghai Jiao Tong University, Shanghai 200240, China; caijf001@sjtu.edu.cn (J.C.)

[3] Shenzhen FBTech Electronics Ltd, Shenzhen 518100, China

[*] Correspondence: jyliu@ahnu.edu.cn (J.L.); chenzhonghua@fbwtt.com (Z.C.); lijinjin@sjtu.edu.cn (J.L.)

[†] These authors contributed equally to this work.

Experimental details

Preparation of ZIF-67 and the derived porous Co_3O_4 nanocages: All the chemicals were purchased from Aladdin Co. Ltd. and used without purification. In the typical process, 0.546 g cobalt nitrate and 0.616 g 2-methyl imidazole were dissolved in 15 mL of methanol solution. After stirring for 15 min magnetically, the solution was centrifugally washed with methanol three times. ZIF-67 was obtained after overnight drying in an oven at 60 °C. The resulting ZIF-67 was annealed in a muffle furnace at a ramping rate of 5 ºC per minute, and was kept at 450 °C for 1 h.

Preparation of Co_3O_4/S-infilled microcapsules: The microcapsules were prepared through a micro-fluidic method. The as-prepared Co_3O_4 nanocages were dispersed in 2% polyvinyl alcohol solution as the internal phase. A certain amount of PVA was added to deionized water and stirred at 70 °C for 2 h as the driving phase. Then, 0.4 g of 2-hydroxy-2-methyl acetophenone and 20 mL of trihydroxymethyl propane ethoxy triacrylate was dissolved in anhydrous ethanol. After the mixture was fully mixed, the liquid was placed overnight in an oven at 60 °C to allow the ethanol completely evaporating. The solution was used as an outer phase. Three syringes were connected to three pumps in the micro-fluidic system. In a coaxial needle, the inner needle was inserted parallel to the outer needle to obtain a coaxial needle. The inside diameter of the inner needle was 0.26 mm; and the outside diameter was 0.49 mm. The inner and outer diameters of the outer needle were 0.84 and 1.27 mm, respectively. The tip of the inner needle was 0.01-0.2 mm longer than the tip of the outer needle. The coaxial needle was assembled by a rubber plug and inserted into the cavity (inner diameter: 19 mm, outer diameter: 25 mm, length: 18 mm). The coaxial needle was directly facing the 0.3 mm hole in the center of the chamber, with a vertical distance of 1.2 mm. The pump that controlled the hydrogel precursor made the hydrogel precursor flowing through the internal needle. At the top of the outlet, the inner phase was wrapped by the outer phase, and the microcapsules were

formed continuously under the shear action of the driving fluid. The flow rates were as follows: inner phase Q_i=3 mL h^{-1}, outer phase Q_o=4 mL h^{-1}, and driving phase Q_f=700 mL h^{-1}. The preparation process was monitored by using a charge-coupled device (CCD) camera (imaging source, DFK-23G274). The capsules were collected and irradiated with ultraviolet light for 6 min to solidify the microcapsule shell. The microcapsules were washed several times with deionized water to get the microcapsules. The resulting sample was calcined at 480 °C for 70 min under nitrogen gas at a ramp rate of 3 °C per min. After that, 0.1 g of the microcapsules and 0.2 g of sulfur powders were mixed in a heat-resistant polytetrafluoroethylene (PTFE) vial filled with argon gas. The vial was sealed and heated at 155 °C for 15 h. At last, the vial was cooled, and the samples were collected for further use.

Characterization: The phase and composition of the samples were measured by X-ray diffraction (Bruker D8 Advance) and X-ray photoelectron spectroscopy (XPS, Escalab 250). The morphology was observed by scanning electron microscopy (SEM, HITACHI S-8100) and high-resolution transmission electron microscopy (HRTEM, HT-7700). Elements were tested on an SEM equipped energy dispersion X-ray spectrometer (EDS, Oxford INCA). Sulfur content was measured using thermogravimetric analysis (TGA, Setaram Labsys EVO SDT Q600). Infrared (IR) spectra were recorded on an IR spectrometer (IR-21IR-21). Raman spectra were measured using a spectroscopic method (Renishaw in Via).

Electrochemical tests: The electrochemical properties of the Co_3O_4/S-infilled microcapsules were studied by a 2032-typed coin cell system. The Li-S battery cathode was made by coating the aluminum foil with a mixture of Co_3O_4/S-infilled microcapsules (70 wt%), carbon black (20 wt%) and polyvinylidene fluoride (10 wt%). The electrode was dried in a vacuum drying oven at 80 °C for 12 h, and then the aluminum foil was cut into 12 mm-diameter disks. All cells were assembled in a glove box (Mikrouna, Super 1220/750/900). The electrolyte consisted of 1, 3-dioxy-pentane and 1, 2-dimethoxy ethane (1:1) mixed with 0.1 M

$LiNO_3$ and 1 M of lithium bistrifluoromethane sulfimide salt. In each cell, the amount of electrolyte was approximately 0.1 mL A battery tester (NEWARE, CT-4008) was used to charge and discharge the Li-S batteries in the potential window of 1.7-2.8 V. Cyclic voltammetry (CV) and electrochemical impedance spectroscopy (EIS) were measured on the same electrochemical workstation (Chenhua, Chi-660E).

Simulation method: In the density functional theory calculations, the exchange correlation function was obtained through a generalized gradient approximation (GGA) algorithm. The interactions between ions and electrons were described by the projected augmented wave (PAW). The geometric configurations of Co_3O_4 and the binding energy of the polysulfides on the surface of Co_3O_4 were calculated by the Perdew–Burke–Ernzerhof (PBE) method. To ensure convergence, the convergence criteria of energy and force were set as $1 \times 10^{-5}\ eV$ and $0.01\ eV/Å$, respectively. The cutoff energy was set as 450eV and a k-point mesh was set as $2 \times 2 \times 2$.

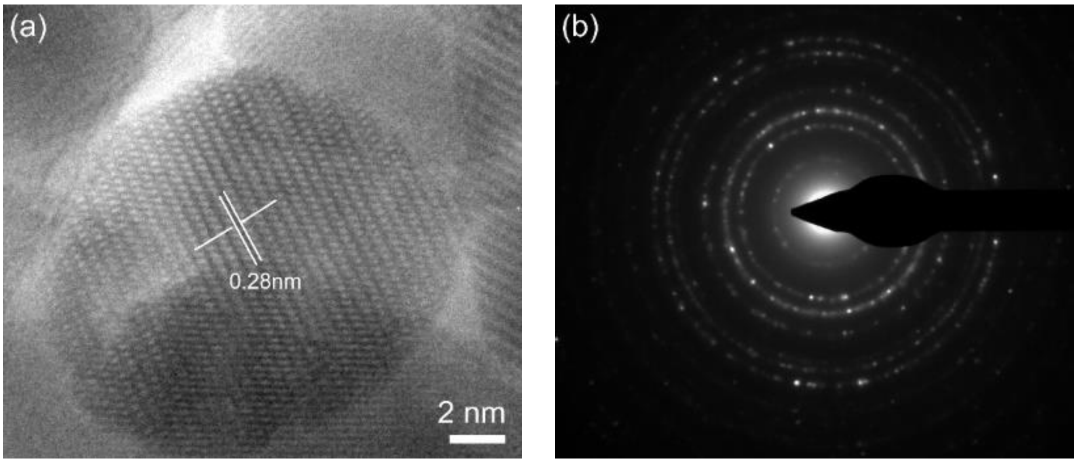

Figure S1. (a) HRTEM image and (b) selective area electron diffraction (SAED) pattern of the porous Co_3O_4.
